# Mesenchymal Stem Cells Yield Transient Improvements in Motor Function in an Infant Rhesus Macaque with Severe Early‐Onset Krabbe Disease

**DOI:** 10.5966/sctm.2015-0317

**Published:** 2016-08-24

**Authors:** Irina A. Isakova, Kate C. Baker, Jason Dufour, Donald G. Phinney

**Affiliations:** ^1^ Donahue's Consulting Inc., New Orleans, Louisiana, USA; ^2^ Department of Veterinary Medicine, Tulane National Primate Research Center, Covington, Louisiana, USA; ^3^ Department of Molecular Therapeutics, The Scripps Research Institute–Scripps Florida, Jupiter, Florida, USA

**Keywords:** Mesenchymal stem cells, Mesenchymal stromal cells, Globoid cell leukodystrophy, Krabbe disease, Nonhuman primates, Cell therapy, Nerve conduction

## Abstract

Krabbe disease, or globoid cell leukodystrophy, is a rare disorder caused by deficient galactosylceramidase activity and loss of myelin‐forming oligodendrocytes, resulting in progressive demyelination and severely impaired motor function. Disease symptoms in humans appear within 3–6 months of age (early infantile) and manifest as marked irritability, spasticity, and seizures. The disease is often fatal by the second year of life, with few effective treatment options. Herein we evaluated the therapeutic potential of mesenchymal stem cells (MSCs) administered intracranially to a 1‐month‐old rhesus macaque diagnosed with severe early‐onset Krabbe disease that displayed neurologic and behavioral symptoms similar to those of human patients. The infant was subjected to physical and neurological behavior examinations and nerve conduction velocity tests to assess efficacy, and outcomes were compared with age‐matched normal infants and Krabbe‐affected rhesus monkeys with late‐onset disease. Changes in major blood lymphocyte populations were also monitored to assess host immune cell responses. MSC administration resulted in transient improvements in coordination, ambulation, cognition, and large motor skills, which correlated with increased peripheral nerve conduction velocities and decreased latencies. Improvements also corresponded to transient increases in peripheral blood lymphocyte counts, but secondary challenge failed to elicit allo‐antibody production. Nevertheless, white cell and neutrophil counts showed dramatic increases, and CD20^+^ B cell counts underwent a precipitous decline at late stages of disease progression. Correlative data linking MSC administration to transient improvements in motor function suggest that MSCs should be evaluated further as an experimental therapy for rare neurodegenerative diseases. Stem Cells Translational Medicine *2017;6:99–109*

**Significance Statement**

This article presents a case history of an infant rhesus macaque diagnosed with severe early‐onset Krabbe disease, for which no current therapy exists. It was demonstrated for the first time that mesenchymal stem cell (MSC)‐based therapy yielded transient but measurable improvements in peripheral nerve conduction, coordination, ambulation, cognition, and large motor skills. Because disease progression in this model is markedly similar clinically and pathologically to the syndrome in humans, these results support additional nonhuman primate studies and future human clinical trials to assess the efficacy of MSCs as experimental therapy for rare neurodegenerative diseases, including Krabbe disease.

## Introduction

Krabbe disease is an autosomal‐recessive disorder resulting from a deficiency of the enzyme galactosylceramidase (GALC) 1, 2, which degrades galactosylceramide and psychosine generated during the process of active myelination. Psychosine is highly toxic to myelin‐forming oligodendrocytes, and its accumulation in nervous tissue results in progressive demyelination. Humans diagnosed with Krabbe disease become symptomatic at 3–6 months of age (infantile‐onset form) and exhibit hypertonia (limb stiffness), extreme irritability, spasticity, seizures, developmental delay, and sensorineural deafness. Krabbe disease may also occur less commonly in adolescence or adulthood (late‐onset form). Diagnosis is typically confirmed by testing for GALC enzyme activity and performing peripheral nerve conduction studies, as conduction velocities are significantly diminished because of loss of compact myelin. Current treatment strategies for Krabbe disease and other lysosomal storage diseases include bone marrow and cord blood cell transplantation 3, 4, enzyme replacement and substrate reduction therapy 5, 6, and small molecule therapy 7. Although these experimental treatments have proved effective for managing some clinical manifestations of a subset of disorders, their benefit in reversing neurological symptoms has been largely ineffective or not firmly established.

Although psychosine accumulation is considered the critical pathogenic mechanism of cell death in Krabbe disease, various studies have demonstrated that inflammation contributes to disease progression. For example, studies conducted in the *Twitcher* mouse, an authentic model of human Krabbe disease 8, indicate that demyelination is invariably accompanied by microglial activation and astrogliosis in the central nervous system (CNS) of these mice 9, 10. Moreover, presentation of major histocompatibility complex (MHC) class II molecules and T cell infiltration 11, 12 as well as increased expression of the proinflammatory cytokines tumor necrosis factor (TNF), interleukin‐6, monocyte chemoattractant protein 1, macrophage inflammatory protein 1α and 1β, and chemokine ligand 5 (also known as RANTES) in the CNS, contribute to disease progression in this model 13 14 15 16. Consistent with these findings, Pasqui et al. 17 found that peripheral blood mononuclear cells (PBMNCs) of human Krabbe‐afflicted patients secreted significantly higher levels of TNF compared with those from healthy subjects, and that TNF production was augmented in the presence of psychosine only in cells obtained from Krabbe patients. These and other studies point to an inflammatory‐immune component to disease progression in Krabbe‐afflicted patients that is likely potentiated by the accumulation of psychosine in the CNS.

Mesenchymal stem cells (MSCs), a type of adult stem cell initially identified in bone marrow, are known to possess potent angiogenic, anti‐inflammatory, and immunomodulatory activities that are largely paracrine in nature 18 19 20 21 22 and are being evaluated in human clinical trials to treat a diverse array of non–skeletal‐related diseases. Consistent with these findings, MSC‐based therapies have yielded benefits in rodent models of storage diseases by restoring deficient enzyme levels, reducing inflammation, and promoting survival of neural cell types 23. We previously demonstrated that MSCs administered via the intracranial route into healthy infant rhesus macaques exhibited low levels of durable cell engraftment and distributed nonrandomly along the brain neuraxis throughout both brain hemispheres, without producing adverse effects on the health or social, behavioral, cognitive, or motor abilities of animals up to 6 months post‐transplant 24 25 26 27. Therefore, based on previous studies showing that MSC‐based therapies ameliorate neuropathology by reducing inflammation in *Twitcher* mice 28, 29, we questioned whether MSCs from a normal allogeneic donor could be therapeutic in a rhesus macaque (*Macaca mulatta*) model of Krabbe disease 30. In this model, newborn rhesus monkeys diagnosed with GALC deficiency appear normal at birth but rapidly develop neurologic and behavioral symptoms similar to those seen in humans, including moderate to severe muscle tremors, impaired muscle coordination resulting in difficult ambulation, irritability, and severely reduced nerve conduction velocities. Depending on the severity of the disease, survival times range from several months to several years. Herein we demonstrate that intracranial administration of MSCs to an infant macaque diagnosed with early‐onset severe Krabbe disease produced a transient but measurable increase in nerve conduction velocities, which corresponded to measurable improvements in coordination, ambulation, cognition, and large motor skills based on physical and behavioral examinations. To our knowledge, this is the first report to correlate improvements in motor function with MSC administration in a large animal model of storage disease.

## Patients and Methods

### Ethics Statement

This study was conducted in accordance with and after approval by the Institutional Animal Care and Use Committee of Tulane University and The Scripps Research Institute–Scripps Florida.

### Animal Subjects

Two infant female rhesus macaques (*M. mulatta)* diagnosed after birth with Krabbe disease via genotyping were assigned to this study over a 4‐year period. One animal was deemed too ill to undergo surgery after initial behavioral and physical assessments were completed and therefore was removed from the study. Four normal infant macaques were used as controls. All infants were housed with their biological mother. Serological testing revealed that the animals were negative for simian immunodeficiency virus, hepatitis B virus, and simian T‐cell leukemia virus. GALC activity in PBMNCs was kindly determined by the laboratory of Dr. Wenger (Jefferson Medical College, Philadelphia, PA). Infants were subjected to physical examination on a biweekly basis, which included assessment of foot and hand grip strength, hindlimb and forelimb firmness, posture, ambulation, and degree of tremor. Animal body weight was measured weekly during the first 2 months of age and then monthly until animals were removed from the study.

### High‐Resolution MHC Genotyping

PBMNCs collected from the Krabbe‐affected transplant recipient (JF65) and an unrelated MSC donor macaque (172‐05) were used to isolate genomic DNA for molecular haplotype determination via allele‐specific polymerase chain reaction and pyrosequencing, using primers specific to a wide range of rhesus macaque Mamu alleles 31. Genotyping was performed at the Genetics Laboratory at the Wisconsin National Primate Research Center (Madison, WI). Genotypes of age‐matched normal infant macaques (IK17, IK18, IK81, and IL60) used as a control group and their respective MSC donors were reported elsewhere 27.

### Neurobehavioral, Developmental, Motor, and Behavioral Assessments

At 7 and 14 days after birth, JF65 was subjected to a 20‐minute battery of tests to document baseline motor function, temperament, and interactive skills using a test adapted for nonhuman primates from the Brazelton Neonatal Behavioral Assessment Scales used with humans 32. The test items included visual orientation and attention span, state control, motor maturity, activity, reflexes and responses, fine and gross motor skills and strength, and temperamental items such as vocalization, fearfulness, self‐quieting abilities, irritability, and distress. Test scores were grouped into four categories: orientation, control, motor maturity, and activity. After surgery, the infant was evaluated in two settings. The first involved the Bayley test of infant development originally developed for use with human infants but modified for nonhuman primates ranging in age from 4 months to 1 year 33. The 10‐minute evaluation consists of problem‐solving, motor, and temperament tests. Briefly, each subject is held on a table by one examiner, and a second examiner places test items in front of the subject and records its response. Twenty‐one separate motor and behavioral test variables were collapsed into three scores for analysis (problem‐solving, motor abilities, and temperament). Tests were conducted monthly at 3–6 months of age. The second setting involved an evaluation of spontaneous behavior. The infant was placed in a socialization/exercise cage (play cage) in groups of four to six individuals on a daily basis. Play cage motor/behavioral activity of infants was recorded monthly, from 2 weeks to 6 months postsurgery. During each 5‐minute observation, 21 aspects of behavior were quantified with a numerical scale in ascending order of maturity and activity 34. These items were grouped into four categories for analysis: large motor, small motor, social, and behavioral state. In all cases, individual scores were compared with a control group of age‐matched normal infant macaques (IK17, IK18, IK81, and IL60).

### Cell Culture

Donor MSCs were isolated from the bone marrow of a male rhesus macaque raised in the virus‐free colony at the New England National Primate Research Center as previously described 24 25 26 and collected at second passage. Peripheral blood collected from JF65 before surgery and at 10, 30, 60, 120, and 150 days postsurgery was depleted of red blood cells using ACK lysis buffer (Thermo Fisher Scientific Life Sciences, Waltham, MA, http://www.thermofisher.com) then subjected to density gradient centrifugation to isolate the mononuclear fraction. Major blood cell subpopulations were quantified in each sample using a Hematology Analyzer Advia 120 (Bayer, Leverkusen, Germany, http://www.bayer.com). Additionally, cells were stained with human antibodies that cross‐react with the following rhesus antigens: CD3, CD4, CD8, CD16, CD20, and HLA‐DR (BD Biosciences, San Jose, CA, http://www.bdbiosciences.com), CD11 (Miltenyi Biotec, Cambridge, MA, http://www.miltenyibiotec.com), counterstained with an appropriate fluorophore‐conjugated secondary antibody, and analyzed using a LSR II Flow Cytometer (BD Biosciences). Unstained cells and cells stained with an isotype control antibody were used as negative controls.

### Surgical Procedures

At 4 weeks of age, JF65 was immobilized with ketamine (10 mg/kg); administered buprenorphine (0.01 mg/kg), acepromazine (0.02 mg/kg), and glycopyrrolate (0.05 mg/kg); and maintained on isoflurane/O_2_ for the duration of the surgery. The anesthetized animal was placed in a stereotactic frame (Kopf Instruments, Tujunga, CA, http://www.kopfinstruments.com/) and administered four injections (25 µl each) of allogeneic MSCs (172‐05; 12.5 × 10^3^ cells/µl) at a rate of 1.2 µl/min. Injections were targeted to the caudate nucleus using stereotactic coordinates determined from magnetic resonance imaging (MRI) scans performed a week before the surgery. In the latter case, the animal was sedated with tiletamine (Telazol) and 60 coronal (1 mm) and 15 sagittal (3 mm) images were obtained using a GE Signa 1.5 Tesla machine (GE Healthcare, Waukesha, WI, http://www.gehealthcare.com). A unique aspect of the animal's dentition was identified and recorded with respect to anterior‐posterior, dorso‐ventral, and lateral coordinates. Postoperatively, JF65 was administered analgesics for 5 days and cephalexin for 2 weeks. At approximately 4 months after MSC administration, JF65 was again examined by MRI to assess qualitatively myelination in the CNS. At 5 months of age, the animal was administered a single dose (1.25 × 10^6^ cells) of donor MSCs (172‐05) via four intramuscular injections (25 µl each, 4 mm apart) into the right thigh. The four age‐matched normal controls were subjected to the same surgical procedure as described previously 27, except that allogeneic MSCs were administered at 6–8 weeks of age. Control animals were also challenged with an intramuscular dose of donor MSCs, but this was not administered until approximately 7–8 months of age.

### Motor Nerve Conduction Studies

JF65 was anesthetized with ketamine (SOP3.1), subepidermal platinum‐coated recording electrodes were placed over the appropriate muscle in the hand or foot, and the nerve was stimulated at distal and proximal sites. The median, ulnar, and tibial motor responses were recorded, and these values were used to calculate nerve conduction velocities as described previously 35. F waves at rest were elicited from the median, ulnar, and tibial nerves by antidromic supramaximal stimulation over the distal nerve and recorded from the same muscles as in the motor conduction study. The minimal latencies of 20 F waves were measured. Electrical stimulation and recording of the evoked responses were performed using a XLTEK Neuromax electromyographic system. Outcomes were compared with existing data from cohorts of normal and Krabbe‐affected macaques with late‐onset disease.

### Immunological Assays

Splenocytes and thymocytes harvested from JF65 were cocultured with donor or third‐party rhesus MSCs, and the extent of cell lysis of the target cells was quantified using the Cyto‐Tox 96 nonradioactive cytotoxicity assay kit (Promega, Madison, WI, http://www.promega.com). Two different quantities of effector cells (5 × 10^4^ and 1 × 10^5^) were cocultured in multiwell plates (0.32 cm^2^) with 1 × 10^4^ MSCs (1:5 or 1:10 ratio) for 24 hours at 37°C. Levels of cytosolic lactate dehydrogenase released into the media were quantified using a colorimetric assay. The percentage of cell‐mediated toxicity was then calculated by comparing effector/target cell reactions to control samples (media alone, effector and target cells alone) using the formula provided by the manufacturer. All samples were run in triplicate. Donor MSCs were also incubated for 1 hour in the presence of 1:2 diluted sera isolated from the transplant recipient or a sham‐operated control animal that did not receive allogeneic MSCs. Cells were washed and stained with the fluorescein isothiocyanate–conjugated antibody specific to the λ‐chain of rhesus immunoglobulins (Miltenyi Biotec), and the extent of cell staining was analyzed by flow cytometry.

### Histology

Animals were euthanized by sedation. Sections of nerve tissue were collected immediately after euthanasia, which occurred at approximately 7 months of age. Tissue was fixed in 10% neutral‐buffered formalin, processed through a graded series of alcohol and xylene, and embedded in paraffin. Hematoxylin and eosin and Luxol fast blue stains were routinely performed on 5‐µm‐thick paraffin sections.

### Statistical Analysis

Differences between experimental groups or time points were evaluated using one‐way analysis of variance, and post hoc analysis was performed using the Tukey method. The critical range for this test was calculated using the formula $\left(M1-M2\right)/\sqrt{\left[{\text{MS}}_{\text{W}}\times \left(1/n\right)\right]}$, where M is the treatment group mean, *MS_W_* is the mean square within group, and *n* is the number of observations per group. Values with α = 0.05 and *p* < .05 were considered statistically significant.

## Results

### Diagnosis and Assessment of Disease Status

**Figure 1:**
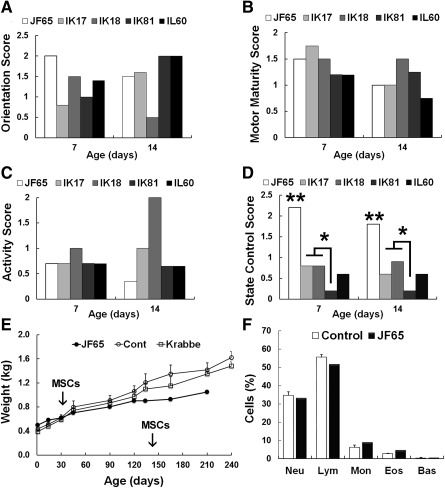
Neurodevelopmental and physical assessments of the Krabbe‐affected infant macaques. **(A–D):** Neurobehavioral assessments of the Krabbe‐affected infant (JF65) and four normal, age‐matched control infants (IK17, IK18, IK81, and IL60) using tests adapted for nonhuman primates from the Brazelton Neonatal Behavioral Assessment scales used for humans. Each 30‐minute test included 42 test items grouped into four clusters to evaluate orientation (attention, visual following) **(A)**, motor maturity (head and trunk posture) **(B)**, activity (quantity of movement) **(C)**, and state control (irritability, struggle, predominant state) **(D)**. ∗∗, *p* < .01 for JF65 compared with control animals; ∗, *p* < .05 for IK17 and IK18 compared with IK81, based on one‐way analysis of variance followed by Tukey ad‐hoc test. **(E):** Body weights (mean ± SD) of JF65 and age‐matched normal (*n* = 10) and mildly symptomatic Krabbe‐affected (*n* = 6) infants plotted as a function of age. **(F):** Complete blood counts for JF65 and four normal, age‐matched control infants from above (mean ± SD) at 2 weeks of age. Abbreviations: Bas, basophil; Cont, control; Eos, eosinophil; Lym, lymphocyte; Mon, monocyte; MSC, mesenchymal stem cell; Neu, neutrophil.

JF65 was diagnosed with Krabbe disease based on genotype analysis and, by several weeks of age, exhibited symptoms consistent with severe early‐onset disease in humans, including a noticeable tremor, weak posture, and impaired ambulation (Table 1). This diagnosis was consistent with the inability to detect measurable levels of GALC activity in the animal's peripheral blood cells (not shown). Neonatal behavioral assessments at 7 and 14 days of age revealed no significant differences with respect to orientation, activity, and motor maturity scores between JF65 and four age‐matched normal infants (IK17, IK18, IK81, and IL60) used as controls, although JF65 did score noticeably lower on the activity test at 14 days of age (Fig. 1A–1C). However, a significant difference [*F*(1,8) = 45.7, *p* = .001] between animals was evident in state control (irritability, agitation, difficult to soothe), and post hoc analysis revealed that scores for JF65 were significantly (*p* < .01) higher than those of each of the age‐matched control infants (Fig. 1D). The neonate was able to eat and showed consistent weight gain by 1 month of age, similar to age‐matched healthy macaques (*n* = 10) raised at the same location (Fig. 1E). Chemistry panels indicated that blood electrolytes, albumin, glucose, creatinine, and liver enzyme levels all fell within the normal range for age‐matched normal infants (not shown). Complete blood cell counts performed at 1 month of age were lower for JF65 compared with age‐matched controls (not shown), but the overall percentage of white cells, neutrophils, platelets, and lymphocytes in blood did not differ between the two groups (Fig. 1F). At 1 month of age, via direct intracranial injection, JF65 was given allogeneic MSCs (172‐05) from an unrelated donor animal partially matched at the Mamu A and E loci (Table 2). The control animals, which were part of a larger study, were also given partially matched MSCs from unrelated donors as reported earlier 27. All animals tolerated the procedure well, and no serious complications were reported. After MSC administration, the growth rate of JF65 remained stable for several months then plateaued at approximately 120 days of age (Fig. 1E), but no significant difference [*F*(2,26) = 0.556, *p* = .58] in body weight was detected between the three groups over the entire 210 days of observation.

**Table 1:** Physical assessments of the Krabbe‐affected infant

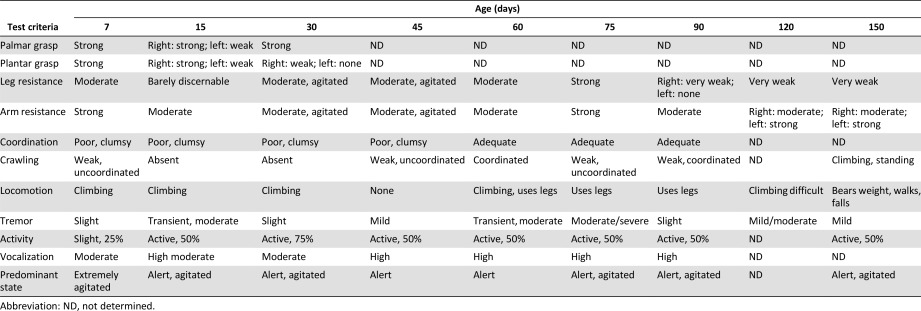

**Table 2:** Major histocompatibility complex phenotype of donor and recipient macaques

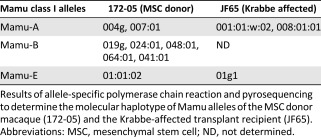

### Host Immune Response to Allogeneic MSC Administration and Disease Progression

**Figure 2:**
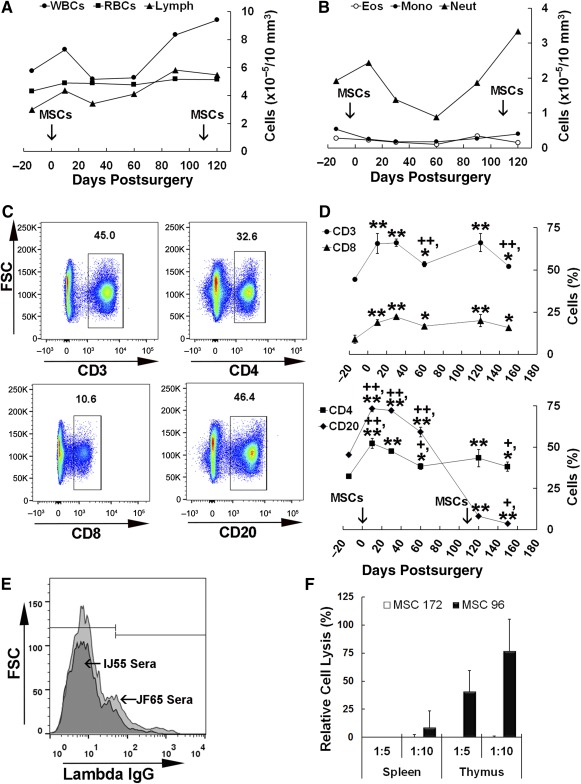
Host immune response to allogeneic MSC administration and disease progression. **(A**,**B):** Number of white/red cells **(A)** and granulocytes/monocytes **(B)** in peripheral blood of JF65 at various times before and after MSC administration. Arrows indicate the timing of MSC administration via the intracranial (day 0) and intramuscular (day 110) routes. **(C):** Representative density plots illustrating the gating strategy used to identify CD3^+^, CD4^+^, CD8^+^, and CD20^+^ lymphocyte populations in peripheral blood of JF65 by flow cytometry. Data are from blood harvested at 2 weeks of age (presurgery). **(D):** Levels of circulating lymphocyte subsets quantified by flow cytometry at various times before and after MSC administration. Plotted values (mean ± SD) are from two separate isolates of peripheral blood taken on the same day. ∗, *p* < .05, ∗∗, *p* < .01 compared with presurgical levels and +, *p* < .05, ++, *p* < .01 compared with 120 days post‐transplant based on one‐way analysis of variance followed by Tukey ad‐hoc test. **(E):** Flow cytometric analysis of donor MSCs preincubated with sera from JF65 or a sham‐operated control animal (IJ55) and stained with a fluorescein isothiocyanate‐conjugated antibody against the λ‐chain of rhesus immunoglobulins. **(F):** Extent of cell lysis (mean ± SD) detected after an overnight incubation of splenocytes and thymocytes isolated from JF65 with the donor MSCs (172) and MSCs from an unrelated third party (96). Abbreviations: Eos, eosinophil; FSC, forward scatter; IgG, immunoglobulin G; Lymph, lymphocyte; Mono, monocyte; MSC, mesenchymal stem cell; Neut, neutrophil; RBC, red blood cell; WBC, white blood cell.

To assess the host immune response to disease pathogenesis and allogeneic MSC administration, we initially performed complete blood cell counts on JF65. As shown in Figure 2A, white blood cell (WBC) and lymphocyte counts transiently increased above presurgical levels at 10 days after MSC administration then returned to presurgical levels by 30 days post‐transplant. This response mimicked that reported previously for age‐matched normal infants given intracranial injections of allogeneic but not autologous MSCs 27 and is indicative of a weak allograft response. However, lymphocyte counts steadily increased from 30 to 90 days post‐transplant then plateaued at 120 days post‐transplant, whereas WBCs steadily increased from 60 to 120 days post‐transplant. In contrast, red blood cell (RBC) counts remained unchanged during the entire time course of observation. Neutrophil counts followed a time course similar to that of WBCs, except they dropped below baseline levels between 30 and 60 days post‐transplant then steadily increased up to 120 days post‐transplant (Fig. 2B). Although neutrophil counts are known to decline in *Twitcher* mice as a function of aging (denervation and degeneration of immune organs) 36, the mechanism responsible for the subsequent spike in neutrophil and WBC counts at late stages of disease progression in JF65 is unknown.

Next, we enumerated specific lymphocyte subsets in peripheral blood from JF65 by flow cytometry (Fig. 2C). This analysis revealed a significant increase in CD3^+^, CD4^+^, CD8^+^, and CD20^+^ subsets 10–30 days after MSC administration compared with baseline levels (Fig. 2D). All populations trended downward at 30–60 days post‐transplant but remained significantly elevated compared with baseline. At 120 days post‐transplant, and 10 days after animals were challenged with a second intramuscular injection of donor MSCs, circulating levels of CD3^+^ and CD4^+^ cells exhibited another transient increase such that levels were significantly higher than at 60 or 150 days post‐transplant. In contrast, CD8^+^ levels remained relatively steady, but CD20^+^ B cell levels declined precipitously at 60–120 days post‐transplant, which did not coincide with either MSC injection and therefore may be indicative of immune atrophy associated with disease progression. Flow cytometric analysis of donor MSCs incubated with sera from JF65 or a sham‐operated control (IJ55) and stained with a rhesus‐specific anti‐immunoglobulin λ‐chain antibody failed to detect the presence of allo‐reactive antibodies with high binding avidity (Fig. 2E). Moreover, splenocytes and thymocytes isolated from JF65 were capable of lysing third‐party MSCs in a dose‐dependent manner in vitro but showed no activity against allogeneic donor MSCs (Fig. 2F). These findings differ from previous results wherein allo‐antibody production was evident in infant macaques administered allogeneic but not autologous MSCs 27. The absence of allo‐antibodies may be caused by tolerance induction or, more likely, reflects the precipitous decline in B cell numbers as a function of disease progression, which may also explain the reduced lytic activity of splenocytes in cell‐based assays.

### MSC Administration Improves Peripheral Nerve Conduction

**Figure 3:**
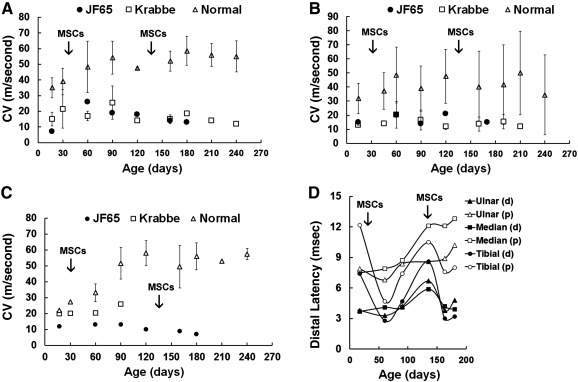
Effect of MSC administration on peripheral nerve conduction velocities and distal latencies. **(A–C):** Conduction velocities as determined for the tibial **(A)**, ulnar **(B)**, and median **(C)** nerves. Plotted values represent mean ± SD for normal infants and infants diagnosed with late‐onset Krabbe disease control groups. **(D):** Distal latencies determined for the proximal and distal ulnar, median, and tibial nerves. Arrows indicate the timing of MSC administration via the intracranial (day 0) and intramuscular (day 110) routes. Abbreviations: CV, conduction velocity; d, distal; MSC, mesenchymal stem cell; p, proximal.

JF65 exhibited extremely low conduction velocities (CVs) for the tibial (7 m/s), ulnar (15 m/s), and median (12 m/s) nerves when evaluated at 2 weeks of age, which was consistent with a diagnosis of severe Krabbe disease (Fig. 3). However, at 1 month after MSC administration, CVs for the tibial nerve, which controls hindlimb movements and posture, increased by 3.7‐fold (7 vs. 28 m/s) (Fig. 3A). This change was consistent with a measurable improvement in coordination, leg and arm resistance, and ambulation based on physical examinations (Table 1). Similarly, CV values for the ulnar nerve also increased by 1.5‐fold over baseline by 30 days post‐transplant and remained at or above pretreatment levels for the duration of the study (Fig. 3B). Although CVs typically increase as a function of age in infant macaques, this is not observed in animals affected with mild Krabbe disease (Fig. 3A–3C). Indeed, tibial nerve CVs of JF65 equaled or exceeded those for the age‐matched group of infants diagnosed with less severe Krabbe disease at a number of time points postsurgery. Moreover, even though values declined from 30 to 150 days post‐transplant, they were still twofold higher at 150 days post‐transplant compared with presurgical levels. In contrast, MSC administration did not produce a measurable increase in median nerve CVs (Fig. 3C). Additionally, there was a marked decrease in distal latencies measured for the tibial nerve (proximal and distal) at 30 days after MSC administration, and minimal decreases were evident in the ulnar but not median nerves (Fig. 3D). However, these values began to increase at 90–135 days of age. Secondary challenge with an intramuscular injection of MSCs did not produce significant increases in nerve CVs, but it did correlate with a transient decrease in distal latencies for all three nerves (Fig. 3D).

### Infant Development and Spontaneous Behavior Assessments

**Figure 4:**
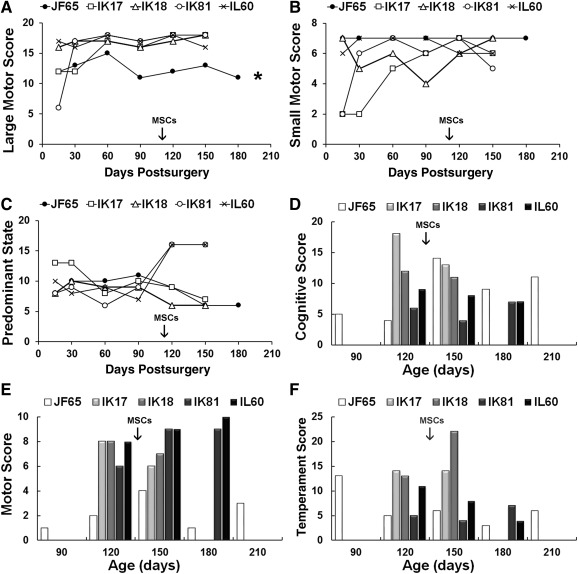
Neurobehavioral assessments of normal and Krabbe‐affected infant macaques. **(A**–**C):** The Krabbe‐affected infant (JF65) and four normal, age‐matched control infants (IK17, IK18, IK81, and IL60) were subjected to focal observations of spontaneous behavior in a novel (play cage) environment that included 13 test items used to define 3 separate rating clusters including large motor skills **(A)**, small motor skills **(B)**, and predominant state **(C)**. Some items not applicable for comparison with Krabbe‐affected infants were not included in determination of scores. **(D**–**F):** Animals from above were also subjected to 10‐minute tests including 15 test items based on the Bayley Scales of Infant Development originally developed for use with human infants. Tests were administered monthly, and items were broken into three rating clusters to measure cognitive ability (orientation and problem‐solving abilities) **(A)**, motor abilities **(B)**, and temperament **(C)**. Arrows indicate the timing of the intramuscular injection of MSCs in JF65. ∗, *p* < .05 for JF65 compared with IK18 and IL60 based on one‐way analysis of variance followed by Tukey ad hoc test. Abbreviation: MSC, mesenchymal stem cell.

JF65 and the age‐matched controls were subjected to spontaneous behavior assessments from 2 weeks to 6 months postsurgery. Notably, only JF655 exhibited tremulousness during the observation sessions. These analyses detected a significant difference [*F*(6,30) = 29.6, *p* = 9.3 × 10^−11^] between animals with respect to overall large motor scores, and post hoc analysis confirmed that scores for JF65 were significantly (*p* < .05) lower compared with IK18 and IL60 (Fig. 4A). Descriptively, scores for JF65 improved from 0 to 2 months postsurgery, declined steadily thereafter, and showed improvement after the animal received the second intramuscular MSC injection. Small motor scores also differed significantly [*F*(6,30) = 6.95, *p* = .002] between animals. Herein, JF65 consistently achieved high scores on these tests, and test results for JF65 differed significantly (*p* < .05) from IK17, who achieved lower scores because of the tendency of this animal to cling to others, resulting in reduced overall activity (Fig. 4B). A significant difference [*F*(6,30) = 7.05, *p* = .002] between animals was also noted for predominant state (irritability, agitation, difficult to soothe), and herein scores for JF65 also descriptively improved from 0.5 to 3 months postsurgery but then steadily declined thereafter (Fig. 4C).

**Figure 5:**
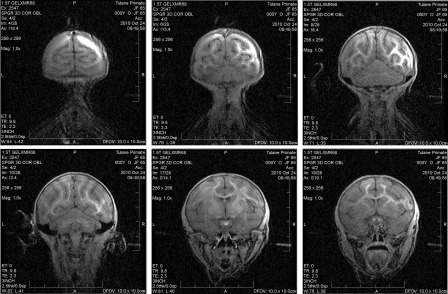
Nuclear magnetic resonance imaging of central nervous system myelin. Coronal images of JF65 at approximately 100 days after direct intracranial mesenchymal stem cell administration to the right brain hemisphere. Images clearly show more pronounced myelination on the injected versus contralateral hemisphere within the occipital white matter, cortical region of the brain, corpus callosum, caudate nucleus, and putamen.

All infants were also subjected to infant development assessments at 3–7 months of age. These tests revealed a significant difference between animals with respect to cognitive [*F*(5,14) = 5.65, *p* = .005], motor [*F*(5,14) = 12.5, *p* = .00009], and temperament [*F*(5,14) = 6.72, *p* = .002] subset scores based on one‐way analysis of variance (Fig. 4D–4F). Descriptively, cognitive subset scores for JF65 at 3 and 4 months of age were well below those measured for normal age‐matched control infants, but then improved dramatically at 5 months of age and remained elevated up to 7 months of age (Fig. 4D). In this regard, improved cognitive skills exhibited by JF65 followed an improvement in ability to navigate the play cage environment, which likely enhanced learning. Both activities may have resulted from increased myelin content of critical brain regions, which was evident based on unweighted MRI scans taken of JF65 at approximately 4.5 months of age (Fig. 5). As expected, motor subset scores were significantly (*p* < .01) higher for each normal infant compared with JF65. In the latter case, scores for JF65 were quite variable but steadily increased from 90 to 150 days of age, which paralleled changes in cognitive ability (Fig. 4E). Finally, temperament subscores were less variable between animals, and tests scores for JF65 showed a steep decline at 3 to 4 months of age and remained low thereafter (Fig. 4F).

### Histopathological Findings

By 5 months of age, the health of JF65 began to deteriorate rapidly such that the animal was deemed unfit for anesthesia, thereby preventing administration of a second intracranial dose of MSCs. Although JF65 was challenged with an intramuscular injection of donor MSCs, this did not significantly delay disease progression, and the animal was humanely euthanized at 7 months of age. Histological analysis of nerve tissue collected at the time of euthanasia confirmed the diagnosis of Krabbe disease. For example, Luxol Fast Blue‐stained sections of sciatic nerve from a normal macaque contained numerous, densely packed, large myelinated fibers (deep blue) that were essentially devoid of surrounding connective tissue ( supplemental online Fig. 1A, 1B). In contrast, tissue from JF65 showed extensive endoneural fibrosis, few if any large myelinated fibers, and the presence of a small number of residual myelinated fibers ( supplemental online Fig. 1C, 1D). Moreover, most of the axons were surrounded by fibrous tissue, which stains pale blue. Electron micrographs of nerve tissue from the right ulna of JF65 further revealed the presence of monocytes with a hollow tubular profile, a Schwann cell with “whorled” structures in its cytoplasm, and an axon with a split myelin sheath ( supplemental online Fig. 1E, 1F). These abnormalities are characteristic of Krabbe disease and confirm the original diagnosis.

## Discussion

A growing number of clinical trials are evaluating MSCs for the treatment of a diverse array of non–skeletal‐related disorders, including those that affect the nervous system. Previously, we conducted a preclinical trial in nonhuman primates (*M. mulatta*) to establish the safety of direct intracranial MSC administration for this specific purpose 24 25 26 27. As an extension of those studies, we now describe outcomes after intracranial administration of allogeneic MSCs in a single infant rhesus macaque diagnosed with early‐onset, severe Krabbe disease 30, 37. Importantly, this large animal model recapitulates to a large degree the disease pathophysiology and neurologic and behavioral symptoms in human infants diagnosed with infantile‐onset Krabbe disease. For example, the affected macaque (JF65) exhibited symptoms as early as 14 days of age, including tremulousness, irritability, impaired motor control, reduced activity, and absence of detectable GALC activity in peripheral blood, which are all symptomatic of severe disease. Nerve conduction velocities measured before MSC administration were also abnormally low and reflected the observed impairments in ambulation and limb coordination and strength. Therefore, outcomes from this study are particularly relevant with respect to translation to the clinical setting.

Despite the severity of its symptoms, JF65 tolerated the intracranial MSC injections well, with no adverse outcomes. Most importantly, the infant responded rather rapidly, with evidence of improved motor control based on physical and behavioral examinations and nerve conduction studies. Although the exact reason for this measurable improvement is unknown, several possibilities exist. First, various studies have shown that MSCs exert trophic effects that improve survival of neural cell types 38 39 40 41, including myelin‐producing oligodendrocytes 42, 43, and also promote maturation of oligodendroglial progenitors toward mature myelin‐producing cells 44, 45. Indeed, based on MRI scans taken at 4.5 months of age, there is a clear indication that the injected versus contralateral hemisphere of the infant's brain contained observably greater amounts of myelin. Although we did not perform measurements to assess the extent of neuro‐inflammation, this also contributes to degeneration in animal models of storage disease and MSCs have been shown to reduce inflammation in mouse models of Krabbe disease (*Twitcher* mice) 28 and chronic experimental autoimmune encephalomyelitis 46. Therefore, the potent anti‐inflammatory effects of MSCs may have salvaged myelin‐producing oligodendrocytes, resulting in increased myelin content of the injected brain hemisphere. Although our study does not explore the specific mechanism of action of MSCs, the observed improvements in ambulation, coordination, large motor control, cognition, and nerve conduction are thoroughly consistent with those reported in other translational models. Nevertheless, the correlative nature of the data do not demonstrate unequivocally that MSC administration was therapeutic in this model. Future studies using placebo‐ and MSC‐treated groups of affected macaques are needed to address this question in a rigorous manner.

Previous studies from our laboratory conducted in healthy infant macaques provided clear evidence that intracranial administration of allogeneic but not autologous MSCs induced an allograft response that involves expansion of natural killer, B, and T cell subsets in peripheral blood, and that its magnitude was dependent on the degree of MHC mismatch between the MSC donor and the transplant recipient 26, 27. This finding was further substantiated in that a secondary challenge with allogeneic donor MSCs induced allo‐antibody production and elevated levels of CD3^−^CD16^+^HLA‐DR^+^ myeloid dendritic cells, which play a major role in peripheral tolerance. In the current study, an effort was made to identify an MSC donor that expressed a similar repertoire of Mamu A1 and Mamu E alleles to minimize the risk of allograft reaction. Nevertheless, we still observed a transient increase in peripheral blood lymphocyte counts several weeks after MSC administration, which mirrored that seen in normal infant macaques after allogeneic MSC administration. However, we were unable to detect evidence of allo‐antibody production in JF65 after secondary antigen challenge. At present, it is unclear whether allo‐antibody production and allo‐reactivity in general were muted or masked by the large increase in WBC and neutrophil counts and decline in CD20^+^ cells in peripheral blood as a function of disease progression. There is evidence for immune organ failure at later stages of disease progression in *Twitcher* mice, which manifests as a decline in WBC but not RBC counts in peripheral blood and decreased cellularity of immune organs, including the spleen and thymus. This inhibits the generation of T and B lymphocytes but spares erythroid cells, resulting in progressive loss of immunocompetence with age 36. Although our findings provide evidence of changes in immune function status, the nature of these changes differs fundamentally from the changes reported in *Twitcher* mice. For example, we observed a precipitous decline in CD20^+^ but not CD3^+^, CD4^+^, and CD8^+^ subsets together with elevated levels of neutrophils and WBCs at late stages of disease. Therefore, atrophy of the spleen and not the thymus may be a peculiar aspect of the disease in macaques. Clearly, if these changes can be validated in a larger cohort of affected macaques, it would represent a fertile area of future study in this model.

## Conclusion

Direct intracranial administration of MSCs to an infant rhesus macaque diagnosed with early‐onset severe Krabbe disease and that was symptomatic at the time of cell administration was well tolerated and resulted in transient but measurable improvements in ambulation, leg and arm resistance, coordination, and nerve CVs. Collectively, these data demonstrate the feasibility of MSCs for use as an experimental therapy to treat storage diseases with neurologic sequelae.

## Author Contributions

I.A.I.: collection and/or assembly of data, data analysis and interpretation, manuscript writing, final approval of manuscript; K.C.B.: conception and design, collection and/or assembly of data, data analysis and interpretation, manuscript writing, final approval of manuscript; J.D.: collection and assembly of data, surgical procedures and animal health monitoring; D.G.P.: conception and design, financial support, data analysis and interpretation, manuscript writing, final approval of manuscript.

## Disclosures of Potential Conflicts of Interest

The author indicated no potential conflicts of interest.

## Supporting information

Supporting Information
