# Supplementary material for: Mesenchymal Stem Cells Yield Transient Improvements in Motor Function in an Infant Rhesus Macaque with Severe Early‐Onset Krabbe Disease
Source: Stem Cells Transl Med. 2016 Aug 24;6(1):99–109. doi: 10.5966/sctm.2015-0317 (PMC5442751; doi:10.5966/sctm.2015-0317)
Supplement: Supplementary file 1 — Supporting Information [file SCT3-6-099-s001.pdf]

Supplemental Figure - Phinney **et al.**

‘

Supplementary Figure 1. Isakova et al. Top

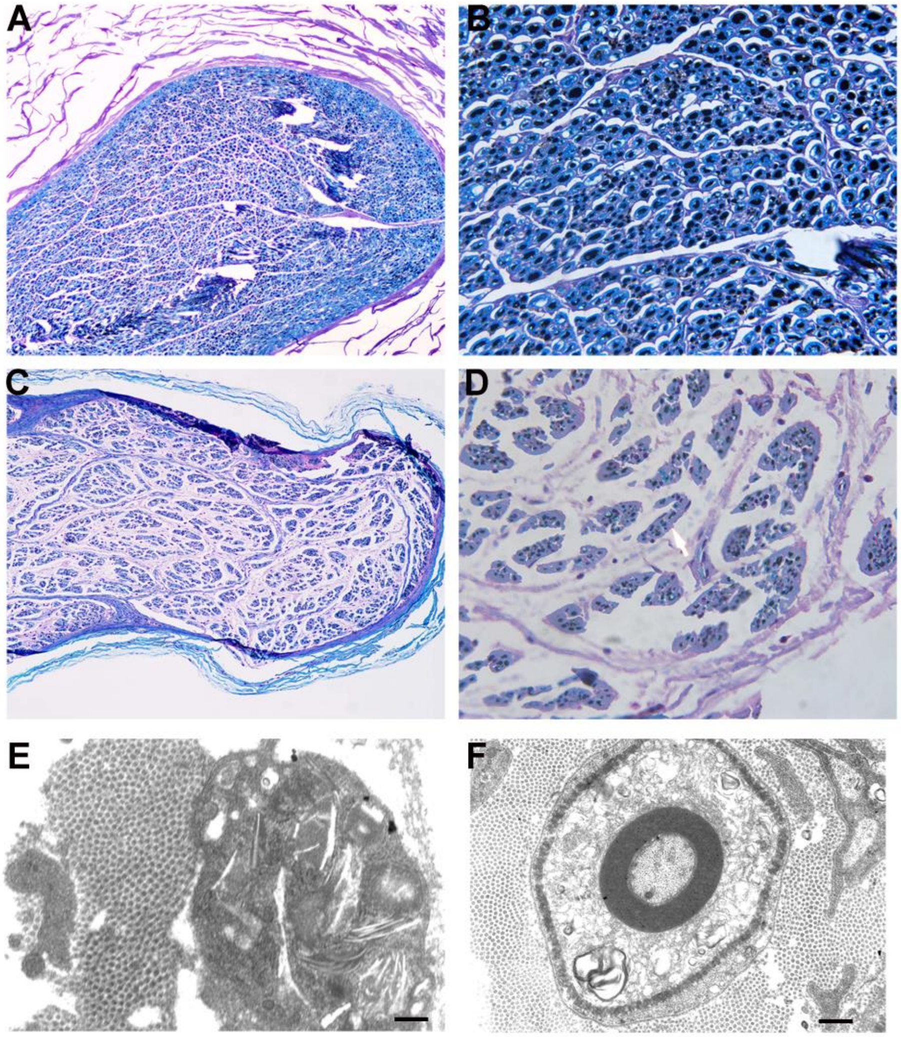

Figure S1. Histopathological evidence of Krabbe disease in JF65. A-D) Photomicrographs of Luxol Fast Blue stained tissue sections of sciatic nerve from an aged-matched normal infant macaque (**A**, **B)** and JF65 (**C**, **D**). Note the appearance of numerous large myelinated (blue) fibers in the control animal whereas nerve tissue from JF65 shows extensive endoneural fibrosis, a marked decrease in large myelinated fibers, and occasional small residual myelinated fibers (white arrow). Moreover, most axons are surrounded by fibrous tissue (pale blue). **E-F**) Electron micrographs of nerve tissue from the right ulna of JF65 showing a monocyte with the characteristic hollow tubular morphology (**E**) and an axon with a split myelin sheath surrounded by collagen fibrils (**F**). Magnification: 10x (A, C), 100x (B, D), 26,100x (E, F). Scale bar in E and F is 500 nm.
